# Concurrence of overt Cushing’s syndrome and primary aldosteronism accompanied by aldosterone-producing cell cluster in adjacent adrenal cortex: case report

**DOI:** 10.1186/s12902-021-00818-2

**Published:** 2021-08-12

**Authors:** Yoshiro Fushimi, Fuminori Tatsumi, Junpei Sanada, Masashi Shimoda, Shinji Kamei, Shuhei Nakanishi, Kohei Kaku, Tomoatsu Mune, Hideaki Kaneto

**Affiliations:** Department of Diabetes, Endocrinology and Metabolism, Kawasaki Medical School, 577 Matsushima, Kurashiki, 701-0192 Japan

**Keywords:** Overt Cushing’s syndrome, Primary aldosteronism, Aldosterone-producing cell cluster, Cytochrome P450 11B1, Cytochrome P450 11B2

## Abstract

**Background:**

Various adrenal disorders including primary aldosteronism and Cushing’s syndrome lead to the cause of hypertension. Although primary aldosteronism is sometimes complicated with preclinical Cushing’s syndrome, concurrence of overt Cushing’s syndrome and primary aldosteronism is very rare. In addition, it has been drawing attention recently that primary aldosteronism is brought about by the presence of aldosterone-producing cell cluster in adjacent adrenal cortex rather than the presence of aldosterone-producing adenoma.

**Case presentation:**

A 67-year-old Japanese female was referred to our institution due to moon face and central obesity. Based on various clinical findings and data, we diagnosed this subject as overt Cushing’s syndrome and primary aldosteronism. Furthermore, in immunostaining for cytochrome P450 (CYP) 11B1, a cortisol-producing enzyme, diffuse staining was observed in tumorous lesion. Also, in immunostaining for CYP11B2, an aldosterone-producing enzyme, CYP11B2 expression was not observed in tumorous lesion, but strong CYP11B2 expression was observed in adjacent adrenal cortex, indicating the presence of aldosterone-producing cell cluster.

**Conclusions:**

We should bear in mind the possibility that concurrence of overt Cushing’s syndrome and primary aldosteronism is accompanied by aldosterone-producing cell cluster in adjacent adrenal cortex.

## Background

It is well known that primary aldosteronism is one of main causes of secondary hypertension. Patients with primary aldosternism more frequently bring about cardiovascular complication compared to those with essential hypertension [1]. However, this is relatively common and curable disease with appropriate diagnosis and treatment. In addion, it was thought that primary aldosteronism was brought about by aldosterone-producing adenoma [2–4]. However, it has been demonstrated recently that primary aldosteronism is closely associated with the presence of aldosterone-producing cell cluster in adjacent adrenal cortex [5–10]. Cushing’s syndrome is another adrenal disorder which autonomously secretes large amounts of cortisol and leads to seconderly hypertension and/or diabetes mellitus. There are two types in this syndrome: preclinical Cushing’s syndrome and overt Cushing’s syndrome accompanied by several characteristic symptoms.

There have been several reports about complication of preclinical Cushing’s syndrome and primary aldosteronism [2–4], but concurrence of overt Cushing’s syndrome and primary aldosteronism is very rare. In addition, it was thought that both aldosterone and cortisol were produced in aldosterone-producing adenoma in subjects with preclinical Cushing’s syndrome and primary aldosteronism [2–4].

Here we show a subject who had overt Cushing’s syndrome and primary aldosteronism. Furthermore, in this subject, we detected cortisol-producing tumor and aldosterone-producing cell cluster in adjacent adrenal cortex, but not in the tumorous lesion, at the same time.

## Case presentation

A 67-year-old Japanese female was referred to our institution due to moon face and central obesity. We thought that she had Cushing’s syndrome from her appearance and measured various markers of the adrenal gland. The results were as follows: adrenocorticotropic hormone (ACTH), < 1.0 pg/mL; cortisol, 11.5 µg/dL; plasma renin activity (PRA), 0.3 ng/mL/h; plasma aldosterone concentration (PAC), 145 pg/mL; aldosterone / renin ratio (ARR), 483.3. Based on these findings, we thought it possible that this patient had concurrence of overt Cushing’s syndrome and primary aldosteronism.

To further examine such abnormalities in this subject, she was hospitalized in our department. On admission, her height, body weight and body mass index were 146.5 cm, 63.0 kg and 29.4 kg/m^2^. Blood pressure, heart rate and body temperature were 150/84 mmHg, 75 /min and 36.1 ℃. Table 1 shows the data on admission. Endocrine markers were as follows: ACTH, < 1.7 pg/mL; cortisol, 10.8 µg/dL; PRA, 0.2 ng/mL/h; PAC, 64.8 pg/mL; ARR, 324. There was no abnormality in glucose metabolism and electrolytes. Liver function and renal function were within normal range.

**Table 1:** Laboratory data in this subject

Peripheral blood (reference range)		Metabolism and endocrine markers		Electrolytes	
RBC	455 × 10^4^ /μL (4.35–5.55)	Plasma glucose	90 mg/dL (73–109)	Sodium	144 mEq/L (138–145)
Hemoglobin	12.6 g/dL (13.7–16.8)	HbA1c	6.2% (4.9–6.0)	Potassium	3.9 mEq/L (3.6–4.8)
WBC	7350 /µL (3300–8600)	LDL cholesterol	147 mg/dL (65–139)	Chloride	106 mEq/L (101–108)
Neutrophils	74.0% (52–80)	HDL cholesterol	60 mg/dL (40–90)	Calcium	9.3 mg/dL (8.8–10.0)
Eosinophils	1.1% (1–5)	Total cholesterol	242 mg/dL (142–248)	Phosphorous	4.6 mg/dL (2.7–4.6)
Basophi	0.4% (0–1)	Triglyceride	150 mg/dL (40–149)	Magnesium	2.1 mg/dL (1.9–2.6)
Monocyte	5.6% (1–6)	TSH	0.96 µU/mL (0.4–6.0)	Urinary storage	
Lymphocytes	18.9% (20–40)	FT3	2.74 pg/mL (2.5–4.2)	Cortisol	73.9 µg/day
Platelet	25.4 × 10^4^ /μL (15.8–34.8)	FT4	1.20 /mL (0.8–1.6)	Aldosterone	7.5 µg/day
Blood biochemistry		ACTH	1.7 pg/mL (7.2–63.3)	Adrenaline	6.5 µg/day
Total protein	7.0 g/dL (6.6–8.1)	Cortisol	10.8 µg/dL (4.5–21.1)	Noradrenaline	105.7 µg/day
Albumin	4.2 g/dL (4.1–5.1)	DHEA-S	31 µg/dL (24–244)	Dopamine	594.8 µg/day
Total bilirubin	0.4 mg/dL (0.4–1.5)	GH	0.10 ng/mL (0–2.47)	Metanephrine	0.03 mg/day
AST	24 U/L (13–30)	IGF-1	86 ng/mL (72–221)	Normetanephrine	0.14 mg/day
ALT	27 U/L (10–42)	PRA	0.2 ng/mL/h (0.2–2.3)	5-HIAA	2.9 mg/day
γ-GTP	19 U/L (13–64)	PAC	64.8 pg/mL (30–159)	VMA	
LDH	234 U/L (124–222)	ARR	324	Concentration	3.4 mg/day
ALP	265 U/L (106–322)	Adrenaline	8 pg/mL (0–100)	Converted value	3.5 mg/mg.Cre
Creatinine	0.65 mg/dL (0.65–1.07)	Noradrenaline	2217 pg/mL (100–450)	HVA	
BUN	15 mg/dL (8–20)	Dopamine	≤ 5 pg/mL (0–20)	Concentration	2.9 mg/day
Uric acid	5.2 mg/dL (3.7–7.8)			Converted value	3.4 mg/mg.Cre

*Abbreviation*s: *RBC* red blood cells, *WBC* white blood cells, *AST* aspartate aminotransferase, *ALT* alanine aminotransferase, *γ-GTP* γ-glutamyl transpeptidase, *LDH* lactate dehydrogenase, *ALP* alkaline phosphatase, *BUN* blood urea nitrogen, *TSH* thyroid-stimulating hormone, *FT3* free triiodothyronine, *FT4* free thyroxine, *ACTH* adrenocorticotropic hormone, *GH* growth hormone, *IGF-1* insulin-like growth hormone, *PRA* plasma renin activity, *PAC* plasma aldosterone concentration, *ARR* aldosterone / renin ratio, *5-HIAA* 5-hydroxyindole acetic acid, *VMA* vanillyl mandelic acid, *HVA* homovanillic acid

**Fig. 1:**
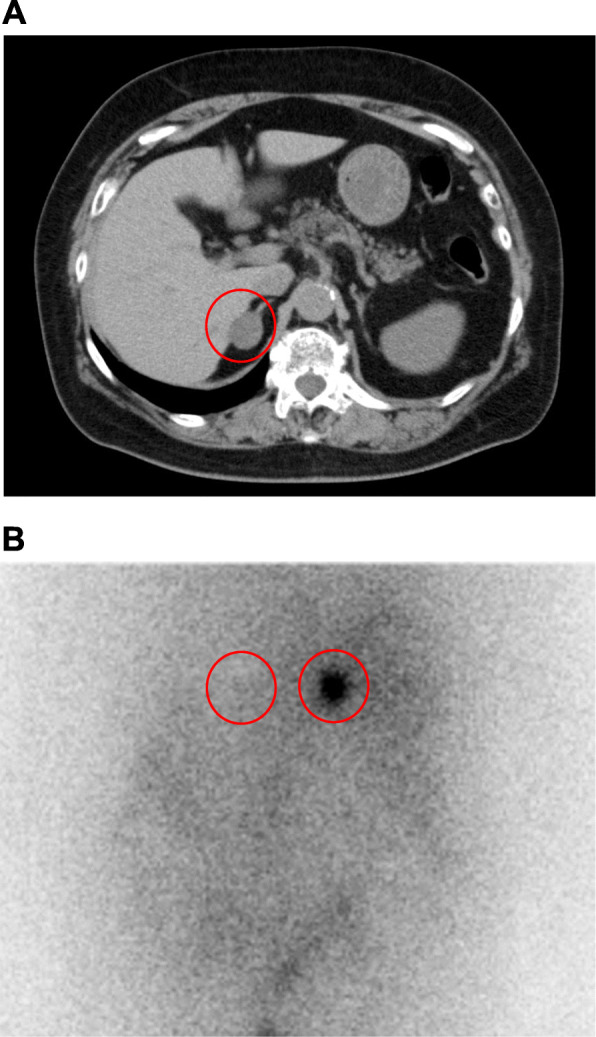
**A** In abdominal computed tomography, tumorous lesion was observed in right adrenal gland. Its size was as large as 25 mm × 22 mm. **B** In adosterol scintigraphy, high accumulation was observed in the same area, whereas there was no accumulation at all in the other side

**Fig. 2:**
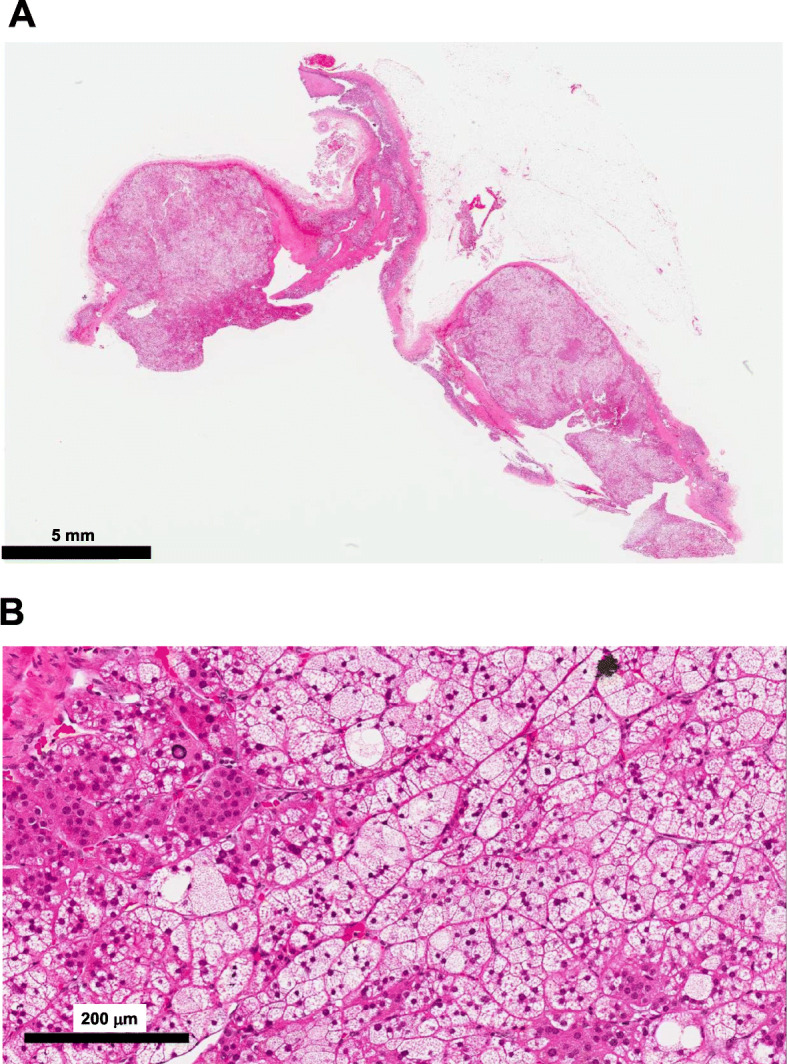
**A** In hematoxylin and eosin (HE) staining, nodular lesions with coat forming and clear boundaries were observed. **B** In high-power field in HE staining, nodular lesion was occupied by foamy and clear cells. Nuclear atypica and abnormal mitosis were not observed, which suggested that there were no malignant findings

**Fig. 3:**
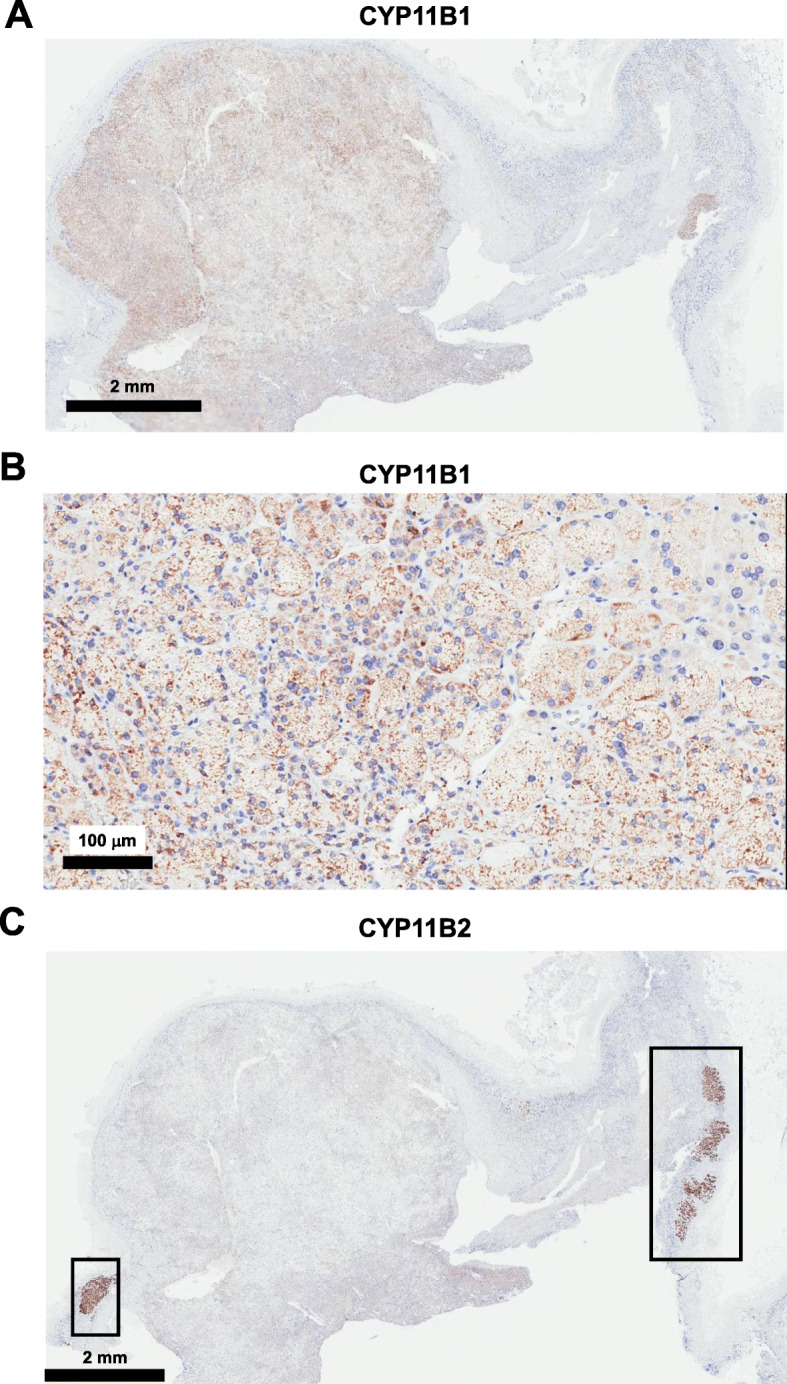
**A** In immunostaining for CYP11B1, a cortisol-producing enzyme, diffuse staining was observed in tumorous lesion. **B** In high-power field in CYP11B1 staining, cytoplasm was diffusely stained. **C** In immunostaining for CYP11B2, an aldosterone-producing enzyme, CYP11B2 expression was not observed in tumorous lesion. However, strong CYP11B2 expression was observed in adjacent adrenal cortex, indicating the presence of aldosterone-producing cell cluster

There was no daily variation in ACTH and cortisol levels (ACTH: 8 am, 1.7 pg/mL, 2 pm, < 0.2 pg/mL, 8 pm, 1.4 pg/mL, 11 pm, 1.0 pg/mL; cortisol: 8 am, 10.8 µg/dL, 2 pm, 11.6 µg/dL, 8 pm, 10.8 µg/dL, 11 pm, 10.0 µg/dL). In dexamethasone suppression test, cortisol level was not reduced (1 mg dexamethasone: ACTH, < 1.0 pg/mL; cortisol 13.2 µg/mL; 8 mg dexamethasone: ACTH, < 1.0 pg/mL; cortisol 12.2 µg/mL). In standing load test, PRA was not increased (PRA: from 0.2 ng/mL/h to 0.2 ng/mL/h; PAC: from 64.8 pg/mL to 81.9 pg/mL). In captopril tolerance test, PRA was not increased (PRA: from < 0.2 ng/mL/h to 0.2 ng/mL/h; PAC: from 89.6 pg/mL to 41.0 pg/mL). In abdominal computed tomography, tumorous lesion was observed in right adrenal gland (25 mm × 22 mm) (Fig. 1A). In adosterol scintigraphy, high accumulation was observed in the right adrenal gland, whereas there was no accumulation in the left adrenal gland (Fig. 1B). It is well known that selective adrenal vein sampling is very important to distinguish unilateral hyperaldosteronism from bilateral one. In this subject, however, overt Cushing’s signs such as moon face and central obesity were observed and the increase of aldosterone level was not drastic. Based on these findings, we thought that the main pathology in this subject was due to Cushing’s syndrome rather than primary aldosteronism and thus we decided not to perform such sampling in this subject. We finally diagnosed this subject as overt Cushing’s syndrome and primary aldosteronism. After then, laparoscopic right adrenalectomy was performed. We further investigated the characteristic of the adrenal tissue. First, in hematoxylin and eosin (HE) staining, nodular lesions with coat forming and clear boundaries were observed, and nodular lesion was occupied by foamy and clear cells. Nuclear atypica and abnormal mitosis were not observed, suggesting that there were no malignant findings (Fig. 2A, B). Second, in immunostaining for cytochrome P450 (CYP) 11B1, a cortisol-producing enzyme, diffuse staining was observed in tumorous lesion (Fig. 3A, B). Third, in immunostaining for CYP11B2, an aldosterone-producing enzyme, CYP11B2 expression was not observed in tumorous lesion (Fig. 3C). Interestingly, however, strong CYP11B2 expression was observed in adjacent adrenal cortex, indicating the presence of aldosterone-producing cell cluster. After the operation, cortisol level was markedly decreased to 0.6 µg/dL, and moon face and central obesity were drastically recovered around 5 months after the operation. In contrast, aldosterone level was not reduced even after the right adrenalectomy. These data suggest that the left as well as right adrenal gland secreted aldosterone although cortisol was secreted only from the right adrenal gland.

## Discussion and conclusions

In this case report, we showed a subject who had overt Cushing’s syndrome and primary aldosteronism at the same time. Furthermore, in pathological analysis, both cortisol-producing tumor and aldosterone-producing cell cluster in adjacent adrenal cortex, but not in tumorous lesion, were clearly detected at the same time in this subject. To the best of our knowledge, this is the first report about concurrence of overt Cushing’s syndrome and primary aldosteronism accompanied by the presence of aldosterone-producing cell cluster in adjacent adrenal cortex. In addion, previously it was thought that both aldosterone and cortisol were produced in aldosterone-producing adenoma [2–4], but new concept has emerged that cause of primary aldosteronism is related to the presence of aldosterone-producing cell cluster in adjacent adrenal cortex [5, 6]. We think that this case report strongly strengthened above-mentioned relatively new concept about the cause of primary aldosteronism.

In addition, adrenal venous sampling is generally recommended, and indeed it is often used for local diagnosis of primary aldosteronism in clinical practice [11]. It has been reported, however, that when tumor diameter is relatively large and accumulation is obviously unilateral in adosterol scintigraphy, adrenal venous sampling is not necessarily performed [12]. In this subject, adrenal tumor size was as large as 25 mm × 22 mm and accumulation was obviously unilateral in adosterol scintigraphy. In addition, this subject had overt Cushing’s syndrome together with moon face and central obesity and strongly hoped for undergoing surgery. Based on these circumstances, we prioritized performance of surgery rather than adrenal venous sampling in this subject.

Taken together, we should bear in mind the possibility that concurrence of overt Cushing’s syndrome and primary aldosteronism is accompanied by aldosterone-producing cell cluster in adjacent adrenal cortex.

## Data Availability

Not applicable.

## Abbreviations

CYP: Cytochrome P450
ACTH: Adrenocorticotropic hormone
PRA: Plasma renin activity
PAC: Plasma aldosterone concentration
ARR: Aldosterone / renin ratio
